# Identification of quantitative trait loci (QTL) for resistance to Fusarium crown rot (*Fusarium pseudograminearum*) in multiple assay environments in the Pacific Northwestern US

**DOI:** 10.1007/s00122-012-1818-6

**Published:** 2012-02-25

**Authors:** G. J. Poole, R. W. Smiley, T. C. Paulitz, C. A. Walker, A. H. Carter, D. R. See, K. Garland-Campbell

**Affiliations:** 1 Department of Crop and Soil Sciences, Washington State University, P.O. Box 6420, Pullman, WA 99164-6420 USA; 2 Columbia Basin Agricultural Research Center, Oregon State University, P.O. Box 370, Pendleton, OR 97801 USA; 3 Department of Plant Pathology, USDA-Agricultural Research Service, P.O. Box 6430, Pullman, WA 99164-6430 USA; 4 Department of Crop and Soil Sciences, USDA-Agricultural Research Service, P.O. Box 6430, Pullman, WA 99164-6430 USA; 5 Present Address: South Australian Research and Development Institute (SARDI), Gate 2b, Hartley Grove, Urrbrae, SA 5064 Australia

## Abstract

Fusarium crown rot (FCR), caused by *Fusarium pseudograminearum* and *F. culmorum*, reduces wheat (*Triticum aestivum* L.) yields in the Pacific Northwest (PNW) of the US by as much as 35%. Resistance to FCR has not yet been discovered in currently grown PNW wheat cultivars. Several significant quantitative trait loci (QTL) for FCR resistance have been documented on chromosomes 1A, 1D, 2B, 3B, and 4B in resistant Australian cultivars. Our objective was to identify QTL and tightly linked SSR markers for FCR resistance in the partially resistant Australian spring wheat cultivar Sunco using PNW isolates of *F. pseudograminerarum* in greenhouse and field based screening nurseries. A second objective was to compare heritabilities of FCR resistance in multiple types of disease assaying environments (seedling, terrace, and field) using multiple disease rating methods. Two recombinant inbred line (RIL) mapping populations were derived from crosses between Sunco and PNW spring wheat cultivars Macon and Otis. The Sunco/Macon population comprised 219 F_6_:F_7_ lines and the Sunco/Otis population comprised 151 F_5_:F_6_ lines. Plants were inoculated with a single PNW *F. pseudograminearum* isolate (006-13) in growth room (seedling), outdoor terrace (adult) and field (adult) assays conducted from 2008 through 2010. Crown and lower stem tissues of seedling and adult plants were rated for disease severity on several different scales, but mainly on a numeric scale from 0 to 10 where 0 = no discoloration and 10 = severe disease. Significant QTL were identified on chromosomes 2B, 3B, 4B, 4D, and 7A with LOD scores ranging from 3 to 22. The most significant and consistent QTL across screening environments was located on chromosome 3BL, inherited from the PNW cultivars Macon and Otis, with maximum LOD scores of 22 and 9 explaining 36 and 23% of the variation, respectively for the Sunco/Macon and Sunco/Otis populations. The SSR markers *Xgwm247* and *Xgwm299* flank these QTL and are being validated for use in marker-assisted selection for FCR resistance. This is the first report of QTL associated with FCR resistance in the US

**Electronic supplementary material:**

The online version of this article (doi:10.1007/s00122-012-1818-6) contains supplementary material, which is available to authorized users.

## Introduction

Fusarium crown rot (FCR) is one of the most persistent soil-borne diseases of dryland wheat (*Triticum aestivum* L.) in the Pacific Northwest (PNW) of the United States, as well as world-wide (Cook 1992; Burgess et al. 2001; Backhouse et al. 2004; Nicol et al. 2007; Smiley et al. 2005a). The disease is caused by a complex of species of which *Fusarium pseudograminearum* (O’Donnell & Aoki) (=*F. graminearum* group I, =*Gibberella coronicola*) and *F. culmorum* (Wm. G. Sm.) Sacc.) are the most significant (Burgess et al. 2001; Smiley et al. 2005a; Nicol et al. 2007). Cook (2001) estimated that soil-borne root diseases cost the US about 10 million tons in wheat production annually. Yield loss due to FCR has been documented to be from 9 to 89%. In the %PNW, yield losses have been documented as high as 35%, with an accepted average of 9%, while world-wide losses exceeding 30% have been reported (Cook 1968, 1992; Kane et al. 1987; Burgess et al. 2001; Dodman and Wildermuth 1987; Klein et al. 1990; Nicol et al. 2007).

A shift in cropping systems toward conservation tillage and greater residue levels has resulted in an increased impact and prevalence of FCR in the PNW and Australia (Smiley et al. 2005a; Paulitz et al. 2002; Burgess et al. 2001). Smiley and Yan (2009) reported a high degree of variation in response to FCR over years and sites and found it difficult to establish reliable FCR tolerance standards for locally released PNW winter wheat cultivars. No cultivar in the PNW has yet been documented with consistent resistance to the disease. Although FCR management in the PNW has focused on cultural practices including time of planting, fertilizer rates, and residue management, host plant resistance has been postulated as the most feasible approach to reducing wheat yield loss due to soil-borne diseases like crown rot (Cook and Veseth 1991; Cook 2001; Paulitz et al. 2002; Smiley and Yan 2009). Due to a lack of documented resistance in PNW cultivars, our approach has been to introgress FCR resistance from Australian cultivars, including Gluyas Early, Gala, Kukri, 2-49 and Sunco, into adapted PNW germplasm (Burgess et al. 2001; Kammholz et al. 2001; Wildermuth et al. 2001; Wallwork et al. 2004; Bovill et al. 2010). The cultivar Sunco, released from the University of Sydney Plant Breeding Institute in 1986, has partial adult plant resistance to FCR (Woolston 2000; Wildermuth et al. 2001; Wallwork et al. 2004) and has been utilized in crosses with PNW breeding lines to improve resistance.

FCR resistance has been documented with a wide range of inoculation method assays and rating systems (including 0–4 scales and severity indices) to quantify disease levels in both seedlings and adult plants (Liddell et al. 1986; Dodman and Wildermuth 1987; Wildermuth and McNamara 1994; Wallwork et al. 2004; Mitter et al. 2006; Nicol et al. 2007; Li et al. 2008). Wallwork et al. (2004) proposed a concept of adult and seedling plant resistance and postulated that more resistant genotypes could be detected in seedling assays, whereas genotypes with partial resistance are better detected in adult plant assays.

To date, most QTL studies have reported QTL in either seedlings or adult plants, but not both. Wallwork et al. (2004) used an outdoor terrace nursery assay to identify FCR resistance QTL derived from the cultivar Kukri. The intent of this assay was to inoculate and assay adult plants in a controlled environment in an attempt to reduce variation typical of field assays due to uneven inoculation and environmental effects. All other studies that have reported FCR resistance QTL utilized greenhouse seedling assays (Collard et al. 2005, 2006; Bovill et al. 2006, 2010; Ma et al. 2009; Li et al. 2010). Ma et al. (2009), Bovill et al. (2010), and Li et al. (2010) reported significant QTL on the long arm of chromosome 3BL inherited from CSCR6, W21MMT70 and Ernie, respectively. Bovill et al. (2010) identified a single significant QTL inherited from Sunco on chromosome 2B (*QCr.usq*-*2B.2*).

Seedling QTL reports have largely used a colonized grain inoculation method and 0–4 summation of leaf sheath symptoms reported by Wildermuth and McNamara (1994) (Collard et al. 2005; Bovill et al. 2006, 2010). Ma et al. (2009) used a seedling dipping inoculation method and rating system reported by Li et al. (2008), whereas Li et al. (2010) used a seedling stem droplet inoculation method reported by Mitter et al. (2006) and a 0–5 severity rating scale (5 = severe disease). Wallwork et al. (2004) reported the only FCR resistance QTL in adult plants using a 0–5 rating scale (0 = no disease; 5 = >75% stem browning).

Our first objective was to identify QTL for resistance to FCR in the cultivar Sunco using two recombinant inbred line (RIL) populations derived from crosses between Sunco and the PNW cultivars Otis and Macon in both seedling (greenhouse) and adult plant (terrace and field) assays. A second objective was to compare heritabilities of FCR resistance and QTL in multiple types of disease assays (greenhouse, terrace, and field) as well as multiple rating systems to identify the most useful system for future screening trials. This research describes novel and significant QTL for FCR resistance inherited from Macon, Otis, and Sunco across two RIL populations in seedling, adult plant terrace and field assaying systems.

## Materials and methods

### Plant materials

Sunco is a hard white spring wheat released from Australia in 1986 with partial resistance to stem rust (*Puccinia graminis* Pers.:Pers. f. sp. *tritici* Eriks. E. Henn.), leaf rust (*P. recondita* Roberge ex Desmaz. f. sp. *tritici*) and FCR (Woolston 2000; Wheat pedigree and identified alleles of genes on-line, 2006, http://genbank.vurv.cz/wheat/pedigree/krizeni2.asp?id=48411). Macon and Otis are PNW-adapted hard white spring wheat cultivars released in 2002 and 2005, respectively (Kidwell et al. 2003, 2006). Otis has high temperature adult plant resistance to stripe rust [*Puccinia striiformis* Westend. f. sp. *tritici* Eriks. (*Pst*)]. Both cultivars have partial resistance to Hessian fly [*Mayotiola destructor* L. (Say)], and dual-purpose end-use quality for noodle and bread making. Two RIL mapping populations were developed, using single seed decent, in greenhouses at the Plant Growth Facility at Washington State University, Pullman, WA. Two hundred and eighteen F_6_:F_7_ RIL were generated from the Sunco/Macon cross. One hundred and fifty-one F_5_:F_6_ RIL were generated from the Sunco/Otis cross. F_6_:F_7_ lines were used in this study as these lines were nearly homozygous at the F_6_:F_7_ generation (~98–99% homozygosity). The mapping population parents, the partially resistant check 2-49, a breeding line from Australia; and the susceptible check Seri, a cultivar developed by The International Maize and Wheat Improvement Center (CIMMYT), were included in all disease screening experiments (Koval and Metakovsky 1988). Each RIL mapping population was grown in the greenhouse for seed increase (non-infected) in 2009 and plant height was recorded at plant maturity and is referred to in this paper as “Greenhouse plant height”. Plant height was also recorded in the field on infected plants from each RIL mapping population during the 2009 field experiment at the Mansfield location and is referred to in this paper as “Field plant height”.

### Phenotypic screening: inoculum preparation

A single *F. pseudograminearum* isolate (006-13) collected near Durfur, Wasco County, Oregon by Smiley and Patterson (1996) was used for screening both RIL mapping populations in several seedling growth room, adult plant terrace, and field assays as it showed the most consistent virulence across several greenhouse optimization studies (Poole 2010). The procedure for inoculum preparation for the growth room and terrace assays was similar to that described by Smiley et al. (2005b), except inoculum was not air dried and was stored at 4°C. Field assays were inoculated with a liquid conidial suspension similar to Nicol et al. (2007). Approximately, 100 g of freshly prepared millet colonized with *F. pseudograminearum* isolate (006-13) was placed in cheesecloth and flushed with deionized distilled water to make approximately 125 mL of conidial solution. Conidial concentrations were checked with a hemocytometer and adjusted to 2.5 × 10^5^ conidia per mL of solution (Mitter et al. 2006; Nicol et al. 2007). Seeds of each line were placed in plastic weigh boats and soaked in 1 mL of the conidial suspension for 1–3 min and dried approximately 2 days prior to planting.

### Phenotypic screening: growth room seedling testing environment

Three runs of the growth room (GR) assay were conducted for each of the RIL populations. The Sunco/Macon RIL were harvested and rated on 7th July 2008 (GR assay 1), 20th September 2008 (GR assay 2), and 20th August 2010 (GR assay 3). The Sunco/Otis RIL were harvested and rated on 15th July 2009 (GR assay 1), 17th September 2009 (GR assay 2), and 30th July 2010 (GR assay 3). Seedlings were planted into a medium comprised 50% sand and 50% peat (Greensmix Sphagnum Peat Moss, Wuapaca Northwoods LLC., Wuapaca, WI, USA) (v/v) in 4 cm diameter × 20.5 cm long cone-tainers (Stuewe and Sons, Corvallis, OR, USA) arranged in plastic trays in a randomized complete block design with at least seven replications. The experiments were conducted in three 27 m^3^ (Conviron, Winnipeg, CA, USA) growth rooms located at the WSU Plant Growth Facility in Pullman, WA. Each growth room could only contain ~1,200 plants. Therefore, for the Sunco/Macon GR assays 1 and 2 the RIL were split into two sets (S) each including common check and parental genotypes. Genotypes were randomized within GR sets. Seedlings were grown at 60/80 (±5)% day/night RH, a 12-h photoperiod, and 25/15°C day/night temperatures until they were rated at 35 days after inoculation. Plants were placed in trays, sub-irrigated as needed, and allowed to dry significantly prior to harvest to stimulate the onset of symptoms. FCR disease symptoms rated on seedlings were the degree of browning of three oldest leaf sheaths and the entire crown region on the basis of several different scales (described below).

### Phenotypic screening: outdoor terrace adult plant testing environment

Three repetitions of the outdoor terrace adult plant screening assay were conducted for the Sunco/Macon RIL and two were conducted for the Sunco/Otis RIL from mid-May through late August during 2008, 2009, and 2010. A single seed was planted into the sand:peat medium described earlier, using larger cone-tainers (6 cm diameter × 24 cm long). The outdoor terrace was divided into four sets (North, South, East, and West) (S) to control spatial variation across the terrace. Parents and check genotypes were included with the RILs in each set. Entries within sets were arranged in a randomized complete block design with ten replications. Approximately, 1.0 g of fertilizer [Osmocote Classic (Scotts Miracle-Gro Co., Marysville, OH, USA) grade 14–14–14 (%N–%P_2_O_5_–%K_2_O)] was added to each cone-tainer.

Following seedling emergence, millet colonized with *F. pseudograminearum* isolate 006-13 was placed approximately 2 cm above the seed and covered with potting mix. Water was immediately applied to the cone-tainers to activate the inoculum and initiate the infection process. Plots were irrigated by an automated overhead sprinkler system on a daily schedule of 1 h per day during emergence, and then at infrequent intervals, as needed, for 2–4 h to encourage root growth. The irrigation was discontinued in late July and disease symptoms were rated in late August on mature plant tissue using the 0–10 scale modified from Nicol et al. (2007) described below.

### Phenotypic screening: field adult plant testing environment

Four field trials were grown during 2009 and 2010 to evaluate the Sunco/Otis and Sunco/Macon RIL mapping populations. In 2009, a single field trial was grown near Mansfield (located at Lat. 48.0 and Long. −119.7) in north central Washington. The Sunco/Otis and Sunco/Macon RIL were planted on May 13 and 20, respectively. In 2010, field trials were grown at three locations and planted on May 6 at Lind (Lat. 47.1 and Long. −118.6) in central WA; at Brewster (Lat. 48.1 and Long. −119.7) in north central WA; and on May 10 at Pullman (Lat. 46.7 and Long. −117.1) in southeastern WA. Following seed inoculation with the liquid conidial suspension method as described above, seed was planted using a deep-furrow hand planting apparatus in 2009. Approximately, 30 seeds were planted in 1.5 m rows, at a depth of 5 cm and a row spacing of 25 cm. In 2010, plots were planted with a Wintersteiger Plotmatic head-row planter (Wintersteiger AG, Reid, Austria) in 1.5 m rows on 15 cm centers. All experiments were arranged as randomized complete blocks, with three replications for Sunco/Otis and two replications for Sunco/Macon in 2009, and three replications of each population during 2010 at all locations. The north central Washington regions near Mansfield and Brewster were typical of dryland wheat growing conditions in the area with a sandy loam soil (Timentwa-Siweeka complex with bedrock; http://websoilsurvey.nrcs.usda.gov) receiving approximately 25 cm of rainfall annually. The soils at Lind and Pullman were Shano silt loam and Thatuna silt loam, respectively. For all sites, after the wheat had senesced, approximately 20 plants were harvested per plot, 5 stems were randomly selected and disease symptoms were rated according to the 0–10 scale described below. Data were expressed as the mean of the five stems per plot.

### Phenotypic screening: comparison of rating assessment methods

Three rating systems were used to quantify disease symptoms in the growth room. The first was a 0–10 scale adapted from the 0–5 rating system described by Nicol et al. (2007). The 0–10 symptom rating system was based on the following scale; 0 = no disease; 1–2 = minor symptoms on crown within the first internode region; 3–4 = obvious symptoms on crown within the first internode region; 5–6 = pronounced symptoms on crown with obvious darkened plant tissue due to infection penetrating to the third leaf; 8–9 = advanced darkened symptoms with severe stunting and near death due to disease infection; 10 = dead plant with severe disease symptoms for FCR severity. In addition to the 0–10 rating system, the Sunco/Macon RIL in growth room seedling assays 1 and 2 were rated by the leaf sheath summation assessment described by Wildermuth and McNamara (1994) and a crown rot severity index (CRI) described by Mitter et al. (2006). The Wildermuth and McNamara leaf sheath sum system involved rating the three oldest seedling leaves on a scale from 0 to 4 (4 = severe disease) with summation to make a total score out of 12. The Mitter et al. (2006) crown rot severity index was calculated as CSI = (length of stem discoloration/seedling height) × (number of leaf sheaths with necrosis). Values for the CSI typically range from 0 to 3.

### Statistical analysis for crown rot disease assays

Separate analyses of the FCR severity ratings were conducted for each population, but methods and models were consistent for the two populations. Experimental units for the growth room and outdoor terrace testing environments were individual plants within a cone-tainer. Experimental units for the field testing environments were individual plots, from which five individual stems were sub-sampled and averaged. Pearson correlations were calculated with Minitab 16 (Minitab Inc., State College, PA, USA) among the FCR severity rating, the crown rot index, and the leaf sheath sum rating systems, and with plant height, as measured on individual plants from the Sunco/Macon population.

The relative importance of sources of variation in the screening experiments was determined using variance component analyses which were conducted separately for each testing environment using the MIXED procedure of SAS System software v9.2 (SAS Institute Inc., Cary, NC, USA). The models all shared the general form: **Y** = **μ** + **Zγ** + **e**, where **Y** is the vector of FCR severity scores, **μ** is the overall mean, **γ** is the vector of random effects of genotypes in each testing environment, **Z** is the associated incidence matrix, and **e** is the vector of experimental errors. The terms **γ** and **e** are considered independent with variance–covariance matrices of **G** and **R**, respectively. The variance–covariance matrix **G** differed between the testing environments, but in all cases covariances were assumed equal to zero. For growth room and terrace testing environments, variance parameters were included for random effects of assay, sets within assays, replicates within sets and assays, and genotypes within sets and assays. For the field testing environment, variance parameters were included for assay, replicates within assays, and genotypes. Experimental error variance was assumed constant (**R** = **I**σ^2^error) for all observations and normally distributed. These assumptions were checked, and in the case of the field data from both populations, modest departure from the assumptions of both constant variance and normality was detected. Therefore, some modest inaccuracy in the estimates of residual variance, standard errors for the variance component estimates, and *z* tests are expected. Assumptions were not substantially violated for the other data sets. Broad sense heritability (*H* ^2^) was estimated within each assay in each testing environment using the formula: *H* ^2^ = Var(*G*)/Var(*P*), where Var(*G*) is the estimated variance of the genotypic effect and Var(*P*) is the estimated variance of the phenotypic effect expressed on a genotype mean basis. The SAS code provided by Holland et al. (2003), modified according to the experimental design of each testing environment, was used for these analyses. Heritabilities also were calculated over all the assays for each testing environment. Least squares means of the genotypes within each assay in each testing environment were calculated using fixed effects versions of the above models in the SAS GLM procedure and *t* tests of the mean differences between the parents were conducted. These least squares means were used in the QTL analyses and to determine genetic correlations among testing environments (see below).

The association of genetic effects among testing environments was determined using genetic correlation with the SAS Mixed procedure, similar to Holland (2006). Our analyses used the following model: **Y** = **μ** + **Xβ** + **Zγ** + **e**, where **Y** is the vector of crown rot severity scores (lsmeans FCR severity for each assay within each testing environment), **β** is the vector of fixed effects of assays within each testing environment, **X** is the associated incidence/design matrix, **γ** is the vector of random effects of genotypes in each testing environment, **Z** is the associated incidence matrix, and **e** is the vector of experimental errors. The terms **γ** and **e** are considered independent with covariance matrices of **G** and **R**, respectively. We considered **G** unstructured for each genotype, with separate variance parameters for each testing environment and heterogeneous covariances between each pair of testing environments. Experimental error variance (**R**) was modeled with heterogeneous variances across testing environment and no covariance between testing environments. Because we used lsmeans for these analyses, the experimental error variance is confounded with genotype by environment interaction. Covariance between testing environments, and by extension phenotypic correlation between testing environments, could not be evaluated in this study because the same environmental conditions cannot be replicated in different testing environments. The assumptions of this method, that residuals are normally, independently, and identically distributed, were tested and confirmed for each model. Wald-type inference tests were used to test if genetic correlations differed from zero.

## Molecular marker analysis

Fresh leaf tissues of each RIL from the F_6_:F_7_ Sunco/Macon and F_5_:F_6_ Sunco/Otis populations, and parents, were collected at the five-leaf stage and stored at −80°C until processing. Genomic DNA was extracted from plant samples using the CTAB method as outlined by Anderson et al. (1992). Two marker systems, DArT and SSR, were utilized to generate a linkage map for each population. DArT genotyping was carried out on both mapping populations by Triticarte Pty. Ltd. (Yarralumla, ACT., Australia; http://www.triticarte.com.au) using the wheat DArT arrays of over 1,200 and 1,500 random markers for Sunco/Macon and Sunco/Otis, respectively. Procedures for hybridization of genomic DNA to the DArT array were conducted using the advanced data set for improved coverage of the D genome according to the description outlined by Akbari et al. (2006).

In addition to DArT markers, 70 polymorphic SSR markers were screened across the parents and both RIL mapping populations. SSR markers were chosen from previously published regions at the distal ends of chromosomes and roughly every 50 cM of the 21 haploid wheat chromosomes according to the linkage map generated by Somers et al. (2004). Template DNA (100 ng/μL) was added to a 11 μL PCR master mix solution for the PCR reaction. The master mix solution consisted of 7.6 μL of nanopure ddH_2_O, 1.2 μL of 10× buffer, 12.4 mM MgCl_2_, 12 mM of dNTPs, 0.6 mM of forward primer (M13 tail), 3 mM of reverse primer, 2.4 mM of appropriate fluorophores, and 0.6 units of *Taq* DNA polymerase. Samples were amplified with an initial denaturation at 94°C for 5 min, followed by 43 cycles of 30 s denaturation at 94°C, 45 s annealing at 50–65°C (depending on primers), and a 1 min extension at 72°C.

SSR analysis was conducted according to PCR conditions as in Röder et al. (1998) with the exception that primers carried the addition of M13 tails for detection on an ABI 3730xl (Applied Biosystems, Foster City, CA, USA) (Oetting et al. 1995). In addition to SSR markers, the reduced height markers *RhtB1a* and *RhtB1b* as well as *RhtD1a* and *RhtD1b* were analyzed on the Sunco/Macon RIL population (Ellis et al. 2002). PCR product data from the ABI 3730xl were visualized and scored using Gene Marker v1.5 software (Soft Genetics LLC, State College, PA, USA). Marker alleles were scored as correlating to the respective parental sized fragments for Macon or Otis (A) and Sunco (B). These scores were used to construct genetic linkage maps in MapMaker v3.0 (Lander et al. 1987) and JoinMap v4.0 (Biometris, Wageningen, The Netherlands, http://www.joinmap.nl) programs according to the methods outlined below (Van Ooijen 2006).

## Linkage map construction and QTL analysis

The DArT markers are dominant whereas the SSR markers are co-dominant. F_7_-derived populations such as these should have some residual heterozygosity which was detected with the SSR markers at about 2%. Segregation ratios of all markers were tested using a Chi-square goodness-of-fit test to a 1:1 ratio at a significance level of *p* = 0.05 and markers that did not fit this ratio were discarded. The final data set comprised 713 markers and 742 markers for the Sunco/Macon population and for the Sunco/Otis population, respectively.

A genome wide linkage map was constructed for each population using MapMaker v3.0. In addition, separate linkage maps were developed only for chromosome 3B for each RIL population. The Kosambi map function was used to generate genetic distances in centiMorgans (cM) between ordered markers (Kosambi 1944). A consensus map for the 3B linkage group was constructed using the “combine groups for map integration” function in JoinMap v4.0, which was followed by the “map” function that resulted in the development of three consensus map result files. The final consensus map was chosen by comparing result maps with previously published wheat consensus maps and the most logical order based on previously publishes linkage maps (Röder et al. 1998; Somers et al. 2004).

The QTL analysis of the association between markers and the lsmeans for FCR severity within each assay and testing environment was conducted for each population using the MapMaker-constructed linkage maps and composite interval mapping (CIM) with WinQTL Cartographer v2.4 (Basten et al. 1997). To detect significant QTL, a critical LOD threshold value of 3.0 was used. For chromosome 3B, CIM was also done using the 3B maps generated in JoinMap v4.0. MapChart v2.2 was used to graphically illustrate significant QTL and linkage groups (Voorrips 2002).

The consistency of marker effects among assays within testing environments was investigated by analyzing the most significant markers within each QTL using separate analyses of variance for each combination of population, marker, and testing environment. All the markers within a QTL that were detected using CIM were analyzed using single-marker analysis with one-way ANOVA and a comparison-wise probability level of *p* < 0.05 to derive a subset of the most significant markers within each QTL. These markers were then analyzed one by one to determine whether significant marker by assay interactions occurred. The SAS MIXED procedure was used with the model: **Y** = **μ** + **Xβ** + **e**, where **Y** is the vector of FCR severity scores, **β** is the vector of fixed effects of screen and marker allele, **X** is the associated incidence/design matrix, and **e** is the vector of experimental errors. Since assays were considered to be repeated measures of each genotype, the covariance structure for **e** was modeled as compound symmetric for each genotype subject. The assumptions of this method, that residuals are normally, independently, and identically distributed, were tested and confirmed for each model. If significant interactions were detected between marker and assay effects, each assay within a testing environment was tested for a significant marker effect individually.

### Diversity panel to analyze polymorphism for *Xgwm247* and *Xgwm299*

A diversity panel of 49 spring and winter wheat cultivars common to the PNW and the USDA-ARS breeding programs at WSU was analyzed to identify polymorphisms for the markers *Xgwm247* and *Xgwm299* which were found to flank a resistance QTL on chromosome 3B, derived from Macon and Otis. DNA was extracted as previously described. Amplicon size was determined as described previously on an ABI 3730xl.

## Results

### Evaluation of disease symptoms for Sunco/Macon

Rating systems were highly correlated. There were strong and significant Pearsons correlations between the 0–10 FCR rating system and the leaf sheath sum rating system (*r* = 0.86; *p* < 0.0001), the 0–10 FCR rating system and the crown rot severity index (*r* = 0.88; *p* < 0.0001), and the leaf sheath sum rating system and the crown rot severity index (*r* = 0.92; *p* < 0.0001). There was a significant Pearson correlation between greenhouse plant height and field plant height (*r* = 0.69; *p* < 0.0001). There was a significant negative Pearson correlation between the terrace mean FCR rating and greenhouse plant height (*r* = −0.27; *p* = 0.001).

Ten phenotyping experiments were conducted to characterize the Sunco/Macon RIL population during 2008, 2009, and 2010. Experiments included three growth room seedling assays, three outdoor terrace adult plant assays, and four field adult plant assays (Table 1). Although the disease ratings for the two parents were frequently not significantly different from each other in the various screening assays, the data for the population exhibited transgressive segregation in all assays (Table 1; see Online Resource 1). The variance component analyses illustrated that non-genetic sources of variation due to assay, replication and residual error were significant in all testing environments (see Online Resource 2). The genotype variance component was significantly different from 0 for the growth room testing and field testing environments but not for the terrace environment.

**Table 1:** FCR disease symptom summary statistics, heritabilities (*H* ^2^), and the peak values for the most significant QTL identified utilizing composite interval mapping (CIM) for each growth room, terrace and field assay for the Sunco/Macon RIL (218 lines) mapping population

Testing environment and trait	Parent^a^			Population statistics^b^							QTL identified				
	Sunco	Macon	*p* value^c^	Min.	Max.	Mean	SD	CV	(*H* ^2^)^d^	SE^d^	Source^e^	Chr.^e^	LOD^e^	A^e^	(*R* ^2^)^e^ (%)
Growth room rate (0–10) assay mean^f^	2.5	3.3	0.003	0.9	5.8	2.8	0.9	90	0.77	0.04	Macon	3BL	22.0	0.50	36
GR rate (0–10) assay 1 July 2008	1.9	2.1	0.89	0.5	7.1	3.0	1.3	86	0.63	0.03	Macon	3BL	12.1	0.60	28
GR rate (0–10) assay 2 September 2008	2.0	3.7	0.12	0.4	6.2	2.9	1.2	114	0.53	0.04	Macon	3BL	11.0	0.50	18
GR rate (0–10) assay 3 July 2010	4.0	3.6	0.66	1.8	8.5	4.6	2.2	49	0.61	0.04	Macon	3BL	12.0	0.70	18
Growth room crown rot index mean^f^	0.1	0.2	0.18	0.0	1.7	0.2	0.2	87	0.71	0.02	Macon	3BL	16.5	0.06	28
GR crown rot index assay 1 July 2008	0.1	0.2	0.14	0.0	2.5	0.2	0.2	119	0.61	0.03	Macon	3BL	13.1	0.06	23
GR crown rot index assay 2 September 2008	0.2	0.2	0.76	0.0	3.0	0.2	0.2	108	0.60	0.03	Macon	3BL	12.1	0.06	20
Growth room leaf sheath sum mean^f^	3.1	3.0	0.89	0.2	11	3.9	2.5	63	0.68	0.02	Macon	3BL	14.5	0.70	25
GR leaf sheath sum assay 1 July 2008	2.0	1.8	0.81	0	12	3.8	3.2	78	0.57	0.04	Macon	3BL	9.3	0.70	19
GR leaf sheath sum assay 2 September 2008	4.0	4.1	0.87	0	12	4.1	3.3	74	0.54	0.04	Macon	3BL	11.4	0.70	19
Terrace mean^f^	4.1	3.4	0.19	1.4	7.0	3.5	1.2	55	0.04	0.11	Macon	1D	2.9	0.22	7
Terrace assay 2008	2.8	3.3	0.30	1.1	4.9	2.9	0.7	66	–^g^	0.07	Macon	3BL	1.3	0.14	3
Terrace assay 2009	5.1	3.3	0.01	1.4	9.7	4.0	1.2	53	0.05	0.09	Sunco	4D	3.0	0.33	7
Terrace assay 2010	5.1	5.4	0.67	1.8	6.5	3.5	1.6	46	0.36	0.06	Macon	3BL	1.0	0.12	2
Field assay mean^f^	1.3	1.3	0.94	0.1	2.7	0.9	0.7	79	0.21	0.11	Macon	3BL	1.3	0.03	1
Field assay Mansfield 2009	2.5	2.3	0.77	0.2	4.2	1.9	0.5	36	0.17	0.11	Macon	3BL	2.1	0.14	4
Field assay Lind 2010	1.2	1.1	0.34	0.1	2.3	0.9	0.6	81	–^g^	–	Macon	3BL	ns^h^	–	–
Field assay Brewster 2010	1.6	1.6	0.66	0.03	3.2	0.8	0.6	75	–	–	Macon	3BL	1.7	0.10	3
Field assay Pullman 2010	1.2	1.2	0.79	0.2	2.7	1.1	0.9	83	–	–	Macon	3BL	1.0	0.09	2
Greenhouse plant height (cm)	71	–	–	27	124	62.6	4.9	7.9	0.94	0.00	Sunco	4B	8.2	4.9	28
Greenhouse plant height (cm)	71	–	–	27	124	62.6	4.9	7.9	0.94	0.00	Macon	4D	4.4	5.0	11
Field plant height (cm)	12	18	0.006	9	23	16.8	2.8	16	0.73	0.03	Sunco	4B	3.6	1.6	19
Field plant height (cm)	12	18	0.006	9	23	16.8	2.8	16	0.73	0.03	Macon	4D	3.8	1.9	9

^a^Parents of the RIL mapping population consisting of 218 individuals

^b^Population parameters of the minimum (min.) and maximum (max.) values, mean, standard deviation (SD), and coefficient of variation (CV) to describe basic statistical parameters for the Sunco/Macon RIL population

^c^ *p* values represent significant differences between the means for Sunco and Macon at *p* < 0.05

^d^Broad sense heritability (*H* ^2^) of each respective growth room, terrace, and field screen calculated by *H* ^2^ = Var(*G*)/Var(*P*) [where Var(*G*) is the variance of the genotypic effect and Var(*P*) is the variance of the phenotypic effect] using the SAS code provided by Holland et al. (2003). Each respective standard error (SE) of variance was calculated for heritability estimates

^e^The inherited cultivar source (Source), chromosomal location (Chr.), likelihood of odds statistic (LOD), additive effect (A), and variation explained (*R* ^2^) for the most significant QTL identified for each respective assay and plant height parameters recorded in the greenhouse and field in 2009

^f^Growth room (GR), terrace, and field assays were carried out in 2008, 2009 and 2010. Ten replications were included in the 2008 2009 terrace assays, while 7 replications were used in the 2009 and 2010 growth room assays. Plants were rated from 0 to 10 according to Nicol et al. (2007); 0 = no disease; 1–2 = minor symptoms on crown within the first internode region; 3–4 = obvious symptoms on crown within the first internode region; 5–6 = pronounced symptoms on crown with obvious darkened plant tissue due to infection; 8–9 = advanced darkened symptoms with severe stunting and near death due to disease infection; 10 = dead plant with severe disease symptoms) for crown rot severity

^g^– missing value. In the case of heritability and standard error, values could not be calculated due to missing values

^h^ns = no suggestive or significant QTL were identified

Significant genotype by assay interactions existed for the growth room seedling trials. The Sunco/Macon growth room assay 3 had significantly more disease symptoms than assay 1, while assays 1 and 2 were not significantly different from each other (Table 1). For the terrace assays, year 2008 had significantly less disease symptoms than years 2009 and 2010 (Table 1). In all years, there were significant differences between sets for the terrace assays (see Online Resource 2). The 2009 Mansfield Field assay had the greatest level of FCR severity (Table 1). Broad sense heritabilities were the greatest for the growth room assays (Table 1). Heritabilities for the terrace assays were essentially equal to 0 in 2008 and 2009 but moderate in 2010. Field heritabilities were low.

Strong genetic correlations were observed between the growth room and the terrace assays for FCR severity (Table 2). The growth room assays had moderate genetic correlations with field assays for FCR severity and field and terrace assays were not significantly genetically correlated. The genetic variance component was the greatest for growth room experiments and least for field experiments with similar trends for the non-genetic variance components (Table 2). In these analysis, the non-genetic variance represents both experimental error and the genotype by environment interaction. These results imply that, although the results of the terrace assays were measured with a great deal of error variance, there were consistent genetic trends among lsmeans for crown rot score from the growth room and terrace testing environments, while the results from the field environments were probably influenced by other environmental effects such as drought, the presence of other diseases etc.

**Table 2:** Genetic correlation coefficients and variance components for the Sunco/Macon and Sunco/Otis RIL populations assessed for crown rot severity across growth room, terrace, and field testing environments

	Growth room^a^	Terrace^b^	Field^c^	Genetic variance^d^	Non-genetic variance^d^
Sunco Macon RIL population					
Growth room^a^	1.00			0.47	1.20
Terrace^b^	0.66 (*p* < 0.0001)^e^	1.00		0.10	0.67
Field^c^	0.39 (*p* = 0.04)^e^	0.14 (*p* = 0.60)^e^	1.00	0.01	0.22
Sunco Otis RIL population					
Growth room^a^	1.00			0.04	0.88
Terrace^b^	–	1.00		–	–
Field^c^	−0.42 (*p* = 0.23)^e^	–	1.00	0.04	0.22

Plants were rated on a 0–10 rating scale adapted from Nicol et al. (2007)

^a^Growth room value is based on 3 growth room screens carried out during 2008, 2009 and 2010

^b^Terrace value is based on 3 terrace screens carried out in 2008, 2009, and 2010

^c^Field value is based on two years of data

^d^Genetic variance and non-genetic variance components were calculated based on means for each assay for each respective growth room, terrace, and field testing environment. The nongenetic component therefore includes genotype by assay interaction, replication and residual sources of variation

^e^ *p* values less than 0.05 were considered significant

### Evaluation of disease symptoms for Sunco/Otis

Nine phenotyping experiments were conducted to characterize the Sunco/Otis RIL population during 2008, 2009 and 2010 (Table 3). Experiments included three growth room assays, two terrace assays, and four field assays. Transgressive segregation was observed for mean FCR severity in the Sunco/Otis population toward susceptibility (Table 3; see Online Resource 1). The rating systems were highly correlated (data not shown). Field plant height and the mean FCR severity across growth room assays were negatively correlated (*r* = −0.16; *p* = 0.04). The FCR severity and range of ratings were generally the lowest for the field assays compared to that of the growth room and terrace assays, as was the case for Sunco/Macon. Similar to Sunco/Macon, the heritabilities were highest in the growth room assays (Table 3). Significant genotype effects were evident across only the growth room and field assays (*p* < 0.0001 and *p* = 0.0002, respectively) (see Online Resource 3) and non-genetic replication and residual error effects were highly significant in all testing environments. Growth room assay 2 had significantly less FCR disease symptoms than assays 1 and 3 (*p* < 0.0001). There were significant differences in the terrace assays between 2009 and 2010 (*p* = 0.004). The 2009 field assay in Mansfield had significantly greater FCR disease symptoms than other locations (*p* < 0.0001). The genetic variance component was much lower for the Sunco/Otis growth room assays compared to that of Sunco/Macon (Table 2). The genetic correlation between growth room and field FCR severity ratings was not significant (*p* = 0.23) (Table 2). We were not able to calculate the genetic correlation between the growth room and terrace due to missing values, but Pearson’s correlations among the two environments were significant but low (0.16; *p* = 0.05).

**Table 3:** FCR disease symptom summary statistics, heritabilities (*H* ^2^), and the peak values for the most significant QTL identified utilizing composite interval mapping (CIM) for each growth room, terrace and field testing environments for the Sunco/Otis RIL (151 lines) mapping population

Testing environment and trait	Parent^a^			Population statistics^b^							QTL identified				
	Sunco	Otis	*p* value^c^	Min.	Max.	Mean	SD	CV	(*H* ^2^)^d^	SE^d^	Source^e^	Chr.^e^	LOD^e^	A^e^	(*R* ^2^)^e^ (%)
Growth room Rate (0–10) mean^f^	3.6	2.5	0.12	2.0	5.2	3.5	0.7	67	0.63	0.04	Otis	3BL	4.0	0.19	10
Growth room assay 1 July 2009	2.7	2.3	0.69	1.7	10.0	3.9	1.1	74	0.37	0.06	Otis	3BL	9.0	0.65	22
Growth room assay 2 September 2009	3.7	3.0	0.52	1.6	5.5	3.1	0.8	59	0.18	0.06	Sunco	4B	2.7	0.23	7
Growth room assay 3 July 2010	3.2	2.5	0.47	1.5	6.8	3.7	1.8	50	0.55	0.05	Sunco	2B	3.2	0.39	8
Terrace mean^f^	5.1	5.1	0.96	2.0	6.5	4.1	1.3	44	0.12	0.26	Sunco	3BS	3.0	0.10	8
Terrace assay 2009	4.6	5.0	0.73	1.5	7.0	4.0	1.1	51	0.31	0.08	Sunco	4B	3.2	0.30	7
Terrace assay 2009	4.6	5.0	0.73	1.5	7.0	4.0	1.1	51	0.31	0.08	Sunco	7A	5.1	0.48	20
Terrace assay 2010	5.6	4.7	0.09	2.6	6.1	4.3	1.6	38	0.49	0.06	Sunco	3BS	4.0	0.11	11
Field assay over environments^f^	1.3	1.1	0.38	0.2	2.8	1.1	0.8	71	0.28	0.15	Otis	3BS	1.0	0.07	3
Field assay 2010 over environments^f^	1.2	1.6	0.38	0.1	3.1	1.1	0.5	46	0.56	0.19	Sunco	3BL	2.5	0.13	7
Field assay Mansfield 2009	1.8	2.5	0.23	1.3	3.5	2.3	0.5	30	0.29	0.10	Otis	3BL	0.8	0.11	2
Field assay Lind 2010	0.3	1.8	0.15	0.2	2.2	0.9	0.7	68	0.25	0.11	Otis	3BL	1.5	0.13	3
Field assay Brewster 2010	1.6	1.6	0.90	0.2	2.7	1.0	0.7	63	0.30	0.09	Sunco	3BL	4.4	0.13	12
Field assay Pullman 2010	1.5	2.6	0.31	0.1	3.4	1.3	1.1	85	-^g^	–	Otis	3BL	1.7	0.12	5

^a^Parents of the RIL mapping population consisting of 151 individuals

^b^Population parameters of the minimum (min.) and maximum (max.) values, mean, standard deviation (SD), and coefficient of variation (CV) to describe basic statistical parameters for the Sunco/Macon RIL population

^c^ *p* values represent significant differences between the means for Sunco and Macon at *p* < 0.05

^d^ Broad sense heritability (*H* ^2^) of each respective growth room, terrace, and field environment calculated by *H* ^2^ = Var(*G*)/Var(*P*) [where Var(*G*) is the variance of the genotypic effect and Var(*P*) is the variance of the phenotypic effect] using the SAS code provided by Holland et al. (2003). Each respective standard error (SE) of variance was calculated for heritability estimates

^e^The inherited cultivar source (Source), chromosomal location (Chr.), likelihood of odds statistic (LOD), additive effect (A), and variation explained (*R* ^2^) for the most significant QTL identified for each respective assay

^f^Growth room (GR), terrace, and field assays were carried out in 2008, 2009 and 2010. Ten replications were included in the 2008 2009 terrace assays, while 7 replications were used in the 2009 and 2010 growth room assays. Plants were rated from 0 to 10 according to Nicol et al. (2007); 0 = no disease; 1–2 = minor symptoms on crown within the first internode region; 3–4 = obvious symptoms on crown within the first internode region; 5–6 = pronounced symptoms on crown with obvious darkened plant tissue due to infection; 8–9 = advanced darkened symptoms with severe stunting and near death due to disease infection; 10 = dead plant with severe disease symptoms) for crown rot severity

^g^Heritability and standard error values could not be calculated due to missing values

### Linkage map construction

A total of 1,225 and 1,544 DArT markers were polymorphic between the parents Sunco and Macon and Sunco and Otis, respectively. In addition 70 SSR markers polymorphic between the parents were analyzed as known chromosomal ‘anchors’. A total of 695 DArT and 18 SSR markers were used to develop the Sunco/Macon linkage map. The final Sunco/Macon linkage map consisted of 328 DArT and 18 SSR markers (346 total) in 39 linkage groups, 27 of which were assigned to each of the 21 haploid chromosomes of wheat. The Sunco/Macon map had a total genetic distance of 1,778 cM. Three linkage groups were assigned to chromosome 1A. Chromosomes 3A, 3B, 7A, and 7B each were comprised two linkage groups. Chromosome 4D was the only under represented linkage group for the Sunco/Macon population containing only four markers. The average distance between markers for this population was 5.1 cM.

A total of 712 DArT and 30 SSR markers were used in the development of the Sunco/Otis linkage map. The final Sunco/Otis linkage map contained 354 DArT and 30 SSR markers in 39 linkage groups, of which 26 were assigned to each of the 21 haploid wheat chromosomes with a total genetic map distance of 2,451 cM. The average distance between markers was 6.3 cM for the Sunco/Otis population. Chromosomes 2B, 3B, 4A, 6A, and 7D each were comprised two linkage groups. In the Sunco/Otis population, there were no unrepresented chromosomes as all 26 linkage groups were assigned to the 21 chromosomes. However, chromosomes 3D, 4D, 5D, and 6D had only 5, 4, 4, and 5 markers within those respective linkage groups assigned to those chromosomes. A total of 108 and 99 markers were mapped to the major linkage group on chromosome 3B for Sunco/Macon and Sunco/Otis, respectively. Our marker screening showed that both Sunco and Otis possessed the *RhtB1b* (*Rht1*) mutation whereas Macon has the *RhtD1b* (*Rht2*) mutation for reduced height.

## FCR resistance QTL identification

### Sunco/Macon

**Fig. 1:**
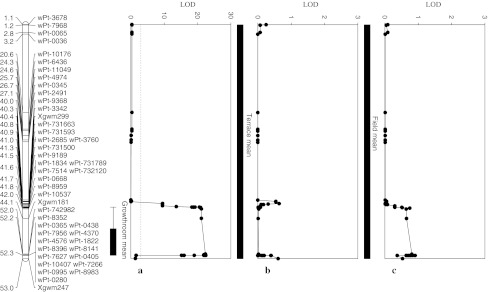
Fusarium crown rot resistance QTL on chromosome 3BL identified in the Sunco/Macon population across three seedling growth room (**a**), terrace (**b**), and field (**c**) testing environments utilizing composite interval mapping

Single-marker analysis revealed significant QTL linked to single markers (*p* < 0.05) on chromosomes 1D, 2B, 3B, 4B, and 4D in seedling growth room and adult plant terrace and field assays (Table 1). Utilizing CIM, significant FCR resistance QTL (LOD > 3.0) were identified on chromosomes 1D, 3B, and 4D (Table 4; Fig. 1). We selected major single markers within each QTL region identified by CIM and ran mixed model analysis to determine whether marker by environmental assay interaction was significant for those QTL. In every case the terrace marker by assay interaction term was non-significant, supporting our identification of a significant marker main effects (see Online Resource 4). With all but one marker for the field assays, the marker by assay interaction terms was non-significant. Marker interaction terms were significant for most markers that had significant main effects for growth room assays in the 3BL region. Significant marker by assay interactions in the one field and growth room assays were not due to cross over interactions, but stemmed from differences in the marker effects due to differences in FCR severity levels between assays 1, 2, and 3.

**Table 4:** Results of the CIM QTL analysis of the significant SSR markers closely linked to *Q*-*crs.wsu*-*3BL* for the respective means of the growth room, terrace, and field testing environments of the Sunco/Macon and Sunco/Otis RIL populations

Marker^a^	Chromosome 3B position	RIL population	Source^b^	Growth room^c^			Terrace^c^			Field^c^		
				LOD	A	*R* ^2^ (%)	LOD	A	*R* ^2^ (%)	LOD	A	*R* ^2^ (%)
wPt-3342	40.3	Sunco/Macon	Macon	19.1	0.5	33	0.02	0.02	<1	0.73	0.03	–
*Xgwm299*	40.4	Sunco/Macon	Macon	18.4	0.5	29	0.03	0.06	1	1.34	0.03	1
wPt-731500	41.3	Sunco/Macon	Macon	22.3	0.6	34	0.38	0.06	1	0.90	0.04	–
*Xgwm181*	44.1	Sunco/Macon	Macon	13.4	0.4	23	0.01	0.06	1	0.98	0.03	1
*Xgwm247*	53.0	Sunco/Macon	Macon	10.0	0.2	2	0.77	0.07	2	0.48	0.03	1
wPt-5390	48.3 (3BS)	Sunco/Otis	Sunco	0.14	−0.05	<1	3.1	−0.35	8	0.94	−0.06	3
*Xwmc777*	52.8 (3BS)	Sunco/Otis	Sunco	0.14	−0.05	<1	1.3	−0.21	4	0.94	−0.06	3
wPt-0021	123.5	Sunco/Otis	Otis	3.1	0.09	7	1.6	0.14	4	0.48	0.04	1
*Xgwm247*	132.5	Sunco/Otis	Otis	3.9	0.18	10	0.53	0.09	1	0.003	0	<1
*Xgwm299*	144.9	Sunco/Otis	Otis	3.0	0.16	7	0.59	0.03	<1	0.10	0	<1
*Xgwm181*	147.5	Sunco/Otis	Otis	0.9	–	2	0.17	0.04	<1	0.03	0	<1

*LOD* likelihood of odds ratio, *A* additive effects of the QTL at each respective marker location, *R* ^*2*^ percent variation described by the QTL at each respective marker locus, *3BS* marker located on the short arm of chromosome 3B

^a^Markers selected for presentation on the basis of significant marker effects in the ANOVA of markers in the QTL region

^b^Parent cultivar contributing the favorable allele

^c^Figures represent means of growth room, terrace, and field environments conducted during 2008, 2009, and 2010

**Fig. 2:**
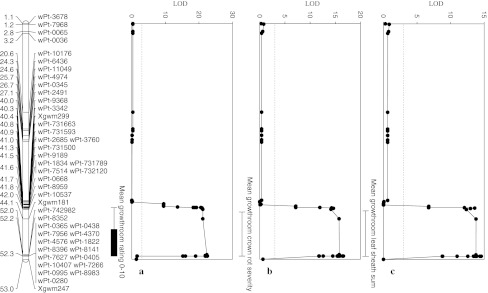
Fusarium crown rot resistance QTL on chromosome 3BL identified across two Sunco/Macon population growth room seedling testing environments utilizing a 0–10 rating system adapted from Nicol et al. (2007) (**a**), a crown rot severity index (Mitter et al. 2006) (**b**), and a leaf sheath summation of symptomatic leaves (Wildermuth and McNamara 1994) (**c**)

A major QTL, *Qcrs.wsu*-*3BL*, inherited from Macon, were identified on chromosome 3BL and was detected in all three growth room seedling assays and to lesser extents in field assays (Tables 1, 4; Fig. 1). The *Qcrs.wsu*-*3BL* QTL are flanked by two microsatellite (SSR) markers, *Xgwm299* and *Xgwm247*, covering a region of 1.8 cM (Fig. 1). The SSR marker *Xgwm299* is located approximately 1.2 cM away from the peak LOD value in the QTL region. All three rating assessment systems successfully identified this major QTL for the Sunco/Macon population in the 3BL region (Fig. 2). Other significant QTL were identified on chromosomes 1D and 4D. The markers *wPt*-*3342* and *wPt*-*731500*, located on chromosome 3BL, were single markers that had significant main effects across all three testing environments (growth room, terrace, and field) (see Online resource 4). The main effects for the SSR markers *Xgwm247*, *Xgwm181*, and *Xgwm299* were significant only in the growth room screens. *Xgwm299* was associated with FCR severity in the field screen at *p* = 0.06 (see Online Resource 4) and was the only marker with suggestive QTL identified from the field in the 3BL QTL location (Table 4).

Four QTL for plant height were identified from greenhouse and field data in the Sunco/Macon population on chromosomes 4B and 4D (Table 1). The plant height QTL on chromosome 4B were inherited from Sunco and was in the region of *RhtB1* (*Rht1*). The plant height QTL on chromosome 4D were inherited from Macon located in the region of *RhtD1* (*Rht2*). The plant height QTL on chromosome 4D were in the same location as significant seedling QTL as rated with the crown rot index and the 0–10 FCR severity rating systems (data not shown).

### Sunco/Otis

**Fig. 3:**
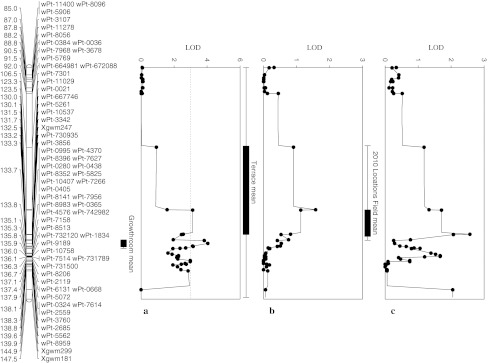
Fusarium crown rot resistance QTL on chromosome 3BL identified in the Sunco/Otis population for the growth room (**a**), terrace (**b**), and field (**c**) testing environment means rated 0–10 utilizing composite interval mapping

**Fig. 4:**
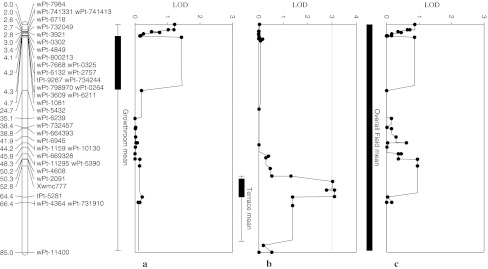
Fusarium crown rot resistance QTL on chromosome 3BS identified in the Sunco/Otis population for the growth room (**a**), terrace (**b**), and field (**c**) environment means rated 0–10 utilizing composite interval mapping

Single marker and CIM analysis revealed significant QTL on chromosomes 2B, 3B, 4B, and 7A with one major QTL on chromosome 3BL inherited from Otis in the Sunco/Otis population (Table 3; see Online Resource 5; Figs. 3, 4). We again ran mixed model analysis to determine whether marker by environment interaction was significant for those QTL. As with the Sunco/Macon population, the marker by assay interaction term was non-significant for most of the terrace and field assays, supporting our identification of significant marker main effects (see Online Resource 5). There was an exception across the terrace assays for two markers on chromosome 7A for markers *wPt*-*5261* and *wPt*-*0021* both in the 3BL QTL region. The terrace marker by assay interactions on 7A were explained by significantly greater levels of disease in individuals with the Otis allele compared to those with the Sunco allele in 2009. Similar to the Sunco/Macon population, the interactions in the growth room were due to non-crossover interactions.

The 3BL QTL region shares 26 of the same DArT markers and 3 of the SSR markers of the Sunco/Macon 3BL QTL region. SSR markers *Xgwm299, Xgwm181,* and *Xgwm247* are linked to this resistance QTL in both RIL populations (Table 3; Figs. 2, 3). Other suggestive QTL were identified in the major 3BL seedling QTL region in the terrace assays as well as the Lind, Brewster, and Pullman field sites, respectively (Table 3). All major seedling QTL in the 3BL region were inherited from Otis. The significant adult plant QTL from the Brewster field location was located in the same chromosome 3BL region as *Qcrs.wsu*-*3BL* prominent in seedling assays, but was inherited from Sunco (Table 3). A significant adult plant QTL, inherited from Sunco, were identified in the 2010 Terrace assay on chromosome 3BS located 57 cM in proximity to *Qcrs.wsu*-*3BL* (Fig. 4). There were not any single markers that had significant main effects across all three testing environments (growth room, terrace, and field) for the Sunco/Otis population. However, markers *wPt*-*667746*, *wPt*-*10130*, *wPt*-*5261*, and *wPt*-*0021* showed significant main effects across growth room and field testing environments (Table 4). SSR markers *Xgwm247*, *Xgwm181*, and *Xgwm299* were only significant across two of the three growth room assays (Table 4). The marker wPt-5390 was located on chromosome 3BS and was highly significant in the terrace testing environments, explained 8% of the variation and was also detected at a lower probability across the field testing environment. The marker *Xwmc777* was also located in the same region of 3BS and was weakly detected in both the terrace and field testing environments. The marker *wPt*-*0021* was significant in the growth room testing environment and was weakly detected across the terrace environment (Table 4).

Another major QTL on chromosome 7A was identified in the 2009 terrace assay (inherited from Otis) and in one of the growth room assays (inherited from Sunco, data not shown) in similar chromosomal regions flanked by the common marker *wPt*-*3702* (Table 3). These QTL were located approximately 31 cM from the SSR marker *Xwmc646*. Other significant QTL, inherited from Sunco, were detected on chromosomes 2B and 4B (LOD scores between 2.7 and 3.2) but were not consistent across assays due to the variable nature of FCR disease symptoms. Because *Qcrs.wsu*-*3BL* derived from Macon and Otis was the major QTL detected in both populations, we developed a consensus map of the region and investigated the allelic diversity of that locus among a diverse sample of wheat cultivars.

### Consensus map

A total of 719 and 726 DArT and SSR markers were imported into JoinMap v4.0 for the development of the Sunco/Macon and Sunco/Otis linkage maps, respectively. Of these markers, 108 (2 groups with 60 and 48 markers) and 101 were assigned to chromosome 3B for Sunco/Macon and Sunco/Otis, respectively. The QTL region on chromosome 3B was in the same region in both populations, located approximately 66 cM from the centromere. The most logical consensus map marker combination for chromosome 3B was a group that resulted in 103 markers covering a distance of 144 cM (see Online Resource 6).

### QTL flanking marker diversity panel

A total of 49 winter and spring wheat cultivars common to the PNW region and the USDA-ARS breeding programs were evaluated for polymorphism for the flanking markers (*Xgwm247* and *Xgwm299*) of *Qcrs.wsu*-*3BL*. Results from the diversity panel showed that the fragment size of Macon and Otis was identical for *Xgwm247* and had a 2 base pair difference for *Xgwm299* (see Online Resource 7). Approximately, 41% of the germplasm analyzed shared the *Xgwm247* locus fragment size of 184 base pairs. Approximately, 28% of the germplasm shared the *Xgwm299* Macon locus (227 bp) and 22% of the germplasm shared the Otis locus (225 bp). The SSR marker *Xgwm299* was the closest marker to *Qcrs.wsu*-*3BL* in the Sunco/Macon population, whereas *Xgwm247* was the closest marker in the Sunco/Otis population. *Xgwm247* is located directly under the largest peak of the 3BL QTL in the Sunco/Otis population and is a potential candidate for validation in segregating populations for potential use in marker-assisted selection. Both the Sunco alleles (for *Xgwm247* and *Xgwm299*) were found only in the cultivar Gala, an Australian hard white spring variety released in 1960 and reported to have partial resistance to FCR. The Australian partially resistant cultivar 2-49 carried alleles similar to that of Macon for the *Qcrs.wsu*-*3BL* loci *Xgwm247* (184 bp) and *Xgwm299* (228 bp). Sunco carried a unique sized fragment for these two marker alleles of 170 bp for *Xgwm247* and 231 bp for *Xgwm299*.

## Discussion

This research reports several significant QTL for FCR resistance inherited from Sunco, Macon, and Otis across growth room, terrace, and field testing environments. The most significant QTL, *Qcrs.wsu*-*3BL,* for FCR resistance identified in the current study were inherited from the PNW cultivars Macon and Otis. The QTL are flanked by the microsatellite SSR markers *Xgwm299* and *Xgwm247*, which may be used for detection of allelic diversity for FCR resistance at this locus.

Bovill et al. (2010) reported a major seedling QTL inherited from Sunco on chromosome 2B (*QCr.usq*-*2B.2*) in a Sunco/2-49 population. In the current study, there was a suggestive QTL on chromosome 2B inherited from Sunco that was identified in the growth room environment in the Sunco/Macon population and a significant QTL on 2B inherited from Sunco in the Sunco/Otis population. However, our chromosome 2B linkage maps and those of Bovill et al. (2010) did not share any markers in common, so currently we cannot confirm that it is the same QTL. Bovill et al. (2010) also reported a minor suggestive (but not significant) QTL inherited from the cultivar Sunco on chromosome 3B in one of three greenhouse seedling assays. In the current study, we found significant QTL inherited from Sunco in the 2010 terrace environment on chromosome 3BS. We identified an adult plant QTL inherited from Sunco in the same region of *Qcrs.wsu*-*3BL* in a single field assay at the Brewster site in 2010. Our results for QTL inherited from Sunco are not consistent and express the highly variable nature of QTL mapping of FCR resistance (Wildermuth and McNamara 1994; Wallwork et al. 2004; Mitter et al. 2006; Smiley and Yan 2009; Bovill et al. 2010).

A major FCR resistance QTL on chromosome 3BL has been reported by others including *Qcr.usq*-*3B.1* (Bovill et al. 2010), inherited from W21MMT70, and *Qcrs.cpi*-*3B* (Ma et al. 2009), inherited from ‘CSCR6’. These could be in a similar location to *Qcrs.wsu*-*3BL* identified in the current study as they share markers in the QTL regions of interest. Although the 3BL QTL reported by Li et al. (2010), inherited from the cultivar Ernie, share the DArT marker, *wPt*-*1834,* with *Qcrs.wsu*-*3BL*, the QTL region also contained *Xwmc471*. In the current study, the marker *Xwmc471* was 67 cM from *Qcrs.wsu*-*3BL* on the Sunco/Macon RIL chromosome 3B map. More in-depth genetic studies and common markers are required for conclusive evidence that these QTL are in the same region.

The major *Qcrs.wsu*-*3BL* identified in the current study was inherited from PNW germplasm that had not been thoroughly screened for FCR resistance prior to this study. The Sunco/Macon mapping population had been developed on the basis of Pythium root rot screening and the Sunco/Otis population selected on preliminary field screening for FCR. Preliminary screening in the greenhouse with local PNW isolates of *F. pseudograminearum* showed that there was a high degree of variability in FCR severity, and the single isolate used for screening in this study (PNW Isolate 006-13) resulted in the most consistent separation of parents and check cultivars. Although preliminary parental screening showed that Sunco carried partial resistance compared to PNW cultivars, results were not consistent (Poole 2010). Transgressive segregation was observed in both populations indicating that the susceptible parent can contribute resistance QTL. Others have also reported significant FCR resistance QTL inherited from the susceptible parent as these cultivars often carry resistance genes for other traits of interest that may contribute to or be linked to FCR resistance (Collard et al. 2005; Bovill et al. 2006; Ma et al. 2009).

An association between plant height and FCR resistance has been reported (Wallwork et al. 2004; Collard et al. 2005; Li et al. 2010). That is, taller plants were more resistant and lacked the *Rht*-*B1b* allele for reduced plant height. In the current study, a significant but low negative correlation between plant height and FCR severity was found in the Sunco/Macon population terrace assays and significant but weak QTL for FCR severity were found on chromosomes 4B and 4D in the Sunco/Macon population. The associations between plant height and FCR resistance are weak and inconclusive in this study.

Rating assessment methods are important to consider in plant breeding studies due to the labor costs associated with accurately rating thousands of plants typically involved in replicated genetic studies. We compared a variety of rating assessment methods previously published by others (Wildermuth and McNamara 1994; Mitter et al. 2006; Nicol et al. 2007). These rating systems were all highly correlated with each other and all rating systems effectively identified *Qcrs.wsu*-*3BL*, as well as other less significant QTL. The 0–10 FCR severity rating system, a simple rating based on observation of overall symptoms of uprooted partially dissected seedlings, was much more efficient, requiring approximately 50% of the time required for the crown rot index and roughly 70% of the time required for the leaf sheath sum rating. This rating system also resulted in the greatest QTL LOD scores for the major QTL identified in this study, *Qcrs.wsu*-*3BL.*

Previous researchers have utilized seedling assays to identify FCR resistance QTL (Collard et al. 2005; Bovill et al. 2006, 2010; Ma et al. 2009; Li et al. 2010), with the exception of Wallwork et al. (2004). This study is the first to include growth room seedling, outdoor terrace adult plant, and field adult plant testing environment data within the same QTL analysis. Heritabilities and genetic variance were greater in the growth room environment compared to adult plant terrace and field environments, due to variation in disease expression and the influence of other environmental factors in field and outdoor environmental conditions noted by others (Wildermuth and McNamara 1994; Wallwork et al. 2004).

The growth room seedling environment had the greatest genetic variance component and was highly heritable, and showed significant but weak genetic correlations with terrace and field assaying systems in the Sunco/Macon population similar to phenotypic correlations reported by Wildermuth and McNamara (1994). However, it appears that significant adult plant and seedling QTL in the current study were located in different genetic regions with different inherited parental sources. The adult plant QTL identified in this study were in the 3BS region, inherited from Sunco, and occurred much less frequently with weaker LOD scores than seedling QTL on 3BL inherited from Macon and Otis. It appears that the concept of seedling and adult plant resistance proposed by Wallwork et al. (2004) could indeed exist. It appears from this study that different assay environments may target different (adult plant or seedling) resistance loci with varying magnitudes of resistance. For example, Wallwork et al. (2004) postulated that partially resistant sources such as Sunco were better detected in adult plant assays. The subject and nature of seedling and adult plant FCR resistance, as well as the validity of currently identified QTL for FCR resistance, require further study through the introgression of current resistance QTL into locally adapted germplasm across different testing environments (growth room, greenhouse, terrace, and field).

The results from the diversity panel showed that many of the PNW genotypes have a similar allele to the *Qcrs.wsu*-*3BL* FCR resistance allele linked to the markers *Xgwm247* and *Xgwm299*. Our trials were inoculated only with *F. pseudograminearum*, but we know from other research that there is a high prevalence of *F. culmorum* as well as mixed populations of both in field environments in the Pacific Northwest (Smiley and Patterson 1996; Poole 2010) (data not shown). Future research should focus on screening of a diverse set of genotypes against a subset of *F. pseudograminearum* and *F. culmorum* isolates collected from a recent FCR survey of the PNW to measure the variation in resistance response at both the seedling and adult plant stage (Poole 2010).

This is the first report of FCR resistance QTL in the US. This research showed that local PNW cultivars Macon and Otis carry a novel and substantial locus for FCR resistance and confirms that unadapted sources of resistance such as that of Sunco are useful to pyramid FCR resistance loci in the US. Further efforts should be made to evaluate more US germplasm for resistance to this disease in both seedling and adult plant field environments. The field screening method could be used as a first assessment of many sources of germplasm, and the seedling method used as a complement. The 0–10 FCR severity scale facilitates rapid screening of many breeding lines. Variability in all of our screening methods is substantial and research into methods of control, including experimental design, additional replication, and inoculation methods, is needed. This result is not unusual in attempts to breed for resistance to *Fusarium* species.

## Electronic supplementary material

Below is the link to the electronic supplementary material.

Supplementary material 1 (DOC 120 kb)

Supplementary material 2 (DOC 28 kb)

Supplementary material 3 (DOC 27 kb)

Supplementary material 4 (PDF 10 kb)

Supplementary material 5 (PDF 9 kb)

Supplementary material 6 (PDF 106 kb)

Supplementary material 7 (PDF 10 kb)
