# Supplementary material for: Identification of quantitative trait loci (QTL) for resistance to Fusarium crown rot (Fusarium pseudograminearum) in multiple assay environments in the Pacific Northwestern US
Source: Theor Appl Genet. 2012 Feb 25;125(1):91–107. doi: 10.1007/s00122-012-1818-6 (PMC3351592; doi:10.1007/s00122-012-1818-6)
Supplement: Supplementary file 1 — Supplementary material 1 (DOC 120 kb) [file 122_2012_1818_MOESM1_ESM.doc]

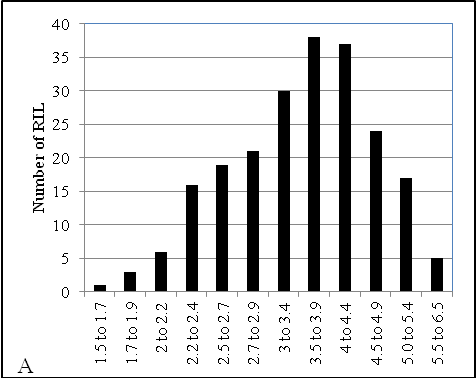

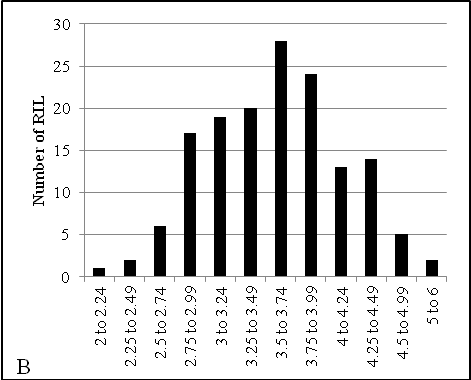


Otis

Sunco

Sunco

Macon


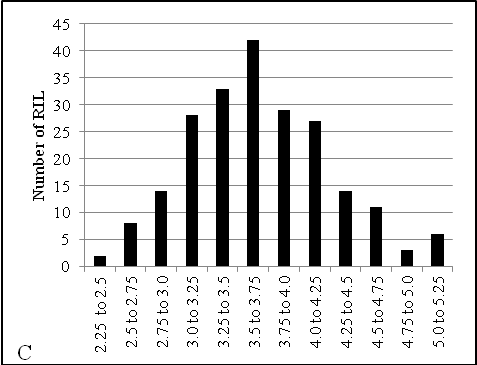

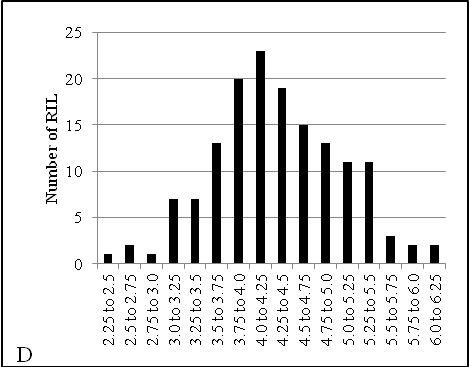


Sunco

Otis

Sunco

Macon


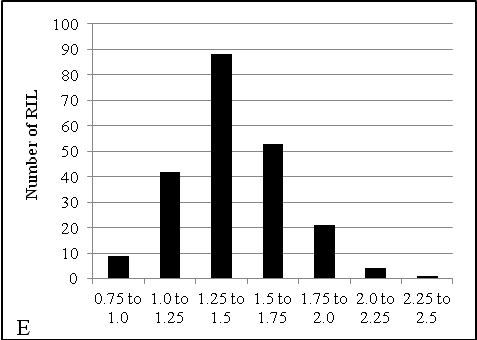

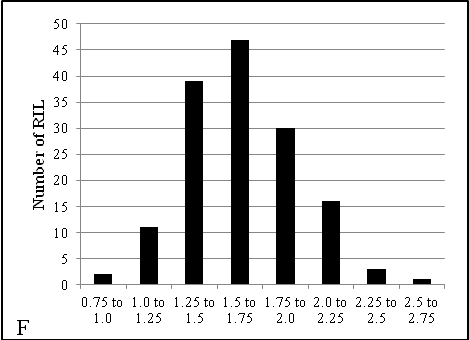


Sunco

Macon

Otis

Sunco

**Online Resource 1.** Histograms of mean Fusarium crown rot severity ratings (0 to 10; 10=severe disease) based on a mean of respective growth room, terrace, or field assays; A = Sunco/Macon growth room seedling assay; B = Sunco/Otis growth room seedling assay; C = Sunco/Macon terrace assay; D = Sunco/Otis terrace assay; E = Sunco/Macon field assay; F = Sunco/Otis field assay
