# Supplementary material for: Identification of quantitative trait loci (QTL) for resistance to Fusarium crown rot (Fusarium pseudograminearum) in multiple assay environments in the Pacific Northwestern US
Source: Theor Appl Genet. 2012 Feb 25;125(1):91–107. doi: 10.1007/s00122-012-1818-6 (PMC3351592; doi:10.1007/s00122-012-1818-6)
Supplement: Supplementary file 2 — Supplementary material 2 (DOC 28 kb) [file 122_2012_1818_MOESM2_ESM.doc]

**Online Resource 2.** Results of the variance components from the analysis of variance for Fusarium crown rot severity across the growth room (GR), terrace, and field testing environments of the Sunco/Macon RIL population

Variance component

Sources of variation estimate Standard error Z-value P-value^a^

Growth room:

Genotype 0.82 0.11 7.24 <0.0001

Assay 0.98 1.11 0.88 0.1895

Assay*Genotype 0.17 0.07 2.59 0.0048

Set^b^ (Assay) 0.13 0.13 1.02 0.1549

Replication (Assay*Set) 0.18 0.06 3.14 0.0009

Residual 5.20 0.12 43.20 <0.0001

Terrace:

Genotype 0.0055 0.025 0.22 0.4133

Assay Year 0.23 0.37 0.62 0.2666

Assay Year*Genotype 0.040 0.033 1.19 0.1176

Set (Assay Year) 0.50 0.26 1.92 0.0276

Replication (Assay*Set) 0.19 0.036 5.31 <0.0001

Residual 3.49 0.06 55.89 <0.0001

Field:

Genotype 0.010 0.0064 1.62 0.0526

Location 0.26 0.22 1.21 0.1136

Location*Genotype 0 . . .

Replication (Location) 0.0057 0.0045 1.25 0.1052

Residual 0.54 0.017 31.27 <0.0001

^a^ P-value considered significant at p=0.05

^b^ Sets = genotypes and checks were randomized within individual growth rooms, or sections of the terrace bed
