# Supplementary material for: Identification of quantitative trait loci (QTL) for resistance to Fusarium crown rot (Fusarium pseudograminearum) in multiple assay environments in the Pacific Northwestern US
Source: Theor Appl Genet. 2012 Feb 25;125(1):91–107. doi: 10.1007/s00122-012-1818-6 (PMC3351592; doi:10.1007/s00122-012-1818-6)
Supplement: Supplementary file 3 — Supplementary material 3 (DOC 27 kb) [file 122_2012_1818_MOESM3_ESM.doc]

**Online Resource 3.** Results of the variance components from the analysis of variance for Fusarium crown rot severity across the growth room (GR), terrace, and field testing environments of the Sunco/Otis RIL population

Variance component

Sources of variation estimate Standard error Z-value P-value^a^

Growth room:

Genotype 0.33 0.061 5.33 <0.0001

Assay 0.082 0.12 0.70 0.2423

Assay*Genotype 0.019 0.048 0.39 0.3473

Set^b^ (Assay) 0.012 0.066 0.18 0.4267

Replication (Assay*Set) 0.20 0.066 2.97 0.0015

Residual 4.23 0.10 40.81 <0.0001

Terrace:

Genotype 0.039 0.086 0.45 0.3272

Assay Year 0 . . .

Assay Year*Genotype 0.18 0.096 1.88 0.0303

Set (Assay Year) 0.32 0.30 1.05 0.1478

Replication (Assay*Set) 0.46 0.12 3.85 <0.0001

Residual 3.46 0.099 35.04 <0.0001

Field:

Genotype 0.042 0.012 3.39 0.0004

Location 0.35 0.30 1.18 0.1186

Location*Genotype 0 . . .

Replication (Location) 0.032 0.018 1.76 0.0390

Residual 0.64 0.023 27.25 <0.0001

^a^ P-value considered significant at p=0.05

^b^ Sets = genotypes and checks were randomized within individual growth rooms, or sections of the terrace bed
