# Supplementary material for: Identification of quantitative trait loci (QTL) for resistance to Fusarium crown rot (Fusarium pseudograminearum) in multiple assay environments in the Pacific Northwestern US
Source: Theor Appl Genet. 2012 Feb 25;125(1):91–107. doi: 10.1007/s00122-012-1818-6 (PMC3351592; doi:10.1007/s00122-012-1818-6)
Supplement: Supplementary file 4 — Supplementary material 4 (PDF 10 kb) [file 122_2012_1818_MOESM4_ESM.pdf]

Online Resource 4. Results from the analysis of variance (ANOVA) of significant markers associated with QTL in the Sunco/Macon RIL population. Main effects and interaction term values represent p-values which were considered significant at p=0.05

		Terrace			Growth room			Field		
Marker[a]	Chromosome	Main Factor	MSI[b]	Screen[c]	Main Factor	MSI[b]	Screen[c]	Main Factor	MSI[b]	Screen[c]
Xwmc429	1D	ns[d]	ns	-	0.05	ns	-	ns	ns	-
Xgwm429	2B	ns	ns	-	0.008	ns	-	ns	ns	-
**wPt-3342**	**3B**	**0.05**	**ns**	**-**	**0.0001**	**0.02**	**all**	**0.02**	**ns**	**-**
Xgwm299	3B	0.24	ns	-	0.0001	0.03	all	0.06	ns	-
wPt-2685	3B	0.06	ns	-	0.0001	0.02	all	0.02	ns	-
wPt-3760	3B	0.09	ns	-	0.0001	0.01	all	0.03	ns	-
**wPt-731500**	**3B**	**0.05**	**ns**	**-**	**0.0001**	**0.008**	**all**	**0.02**	**0.05**	**1**
wPt-9189	3B	0.06	ns	-	0.0001	0.01	all	0.02	ns	-
wPt-1834	3B	0.09	ns	-	0.0001	0.008	all	0.03	ns	-
wPt-731789	3B	0.09	ns	-	0.0001	0.006	all	0.05	ns	-
wPt-7514	3B	0.07	ns	-	0.0001	0.008	all	0.056	ns	-
wPt-732120	3B	0.08	ns	-	0.0001	0.01	all	0.02	ns	-
wPt-0668	3B	ns	ns	-	0.0001	0.01	all	0.02	ns	-
wPt-8959	3B	0.08	ns	-	0.0001	ns	ns	0.03	ns	-
wPt-10537	3B	ns	ns	-	0.0001	0.03	all	0.02	ns	-
Xgwm181	3B	0.3	ns	-	0.0001	ns	-	0.04	ns	-
**wPt-742982**	**3B**	**0.04**	**ns**	**-**	**0.0001**	**ns**	**-**	**0.06**	**ns**	**-**
Xgwm247	3B	0.11	ns	-	0.0001	ns	-	ns	ns	-
Xgwm251	4B	0.45	ns	-	ns	ns	-	ns	ns	-
wPt-3058	4D	ns	ns	-	0.0032	ns	-	ns	ns	-
Xcfd084	4D	ns	ns	-	0.05	0.008	3	ns	ns	-
Xwmc285	4D	0.08	ns	-	ns	ns	-	0.06	ns	-

[a] Individual markers were selected that were most closely associated with significant QTL across all three screening environments

[b] MSI = Marker x screen interaction term

[c] Screen = Screen number where the marker interaction was significant

[d] ns = not significant. P-value was not significant at p=0.05
