# Supplementary material for: Identification of quantitative trait loci (QTL) for resistance to Fusarium crown rot (Fusarium pseudograminearum) in multiple assay environments in the Pacific Northwestern US
Source: Theor Appl Genet. 2012 Feb 25;125(1):91–107. doi: 10.1007/s00122-012-1818-6 (PMC3351592; doi:10.1007/s00122-012-1818-6)
Supplement: Supplementary file 5 — Supplementary material 5 (PDF 9 kb) [file 122_2012_1818_MOESM5_ESM.pdf]

Online Resource 5. Results from the analysis of variance (ANOVA) of significant markers associated with QTL in the Sunco/Otis RIL population. Main effects and interaction term values represent p-values which were considered significant at p=0.05

		Terrace			Growth room			Field		
Marker[a]	Chromosome	Main Factor	MSI[b]	Screen[c]	Main Factor	MSI[b]	Screen[c]	Main Factor	MSI[b]	Screen[c]
wPt-665228	1A	0.06	ns	-	ns	ns	-	ns	ns	-
wPt-667746	1A	ns	ns	-	0.0001	0.001	1	0.03	0.01	3,4
wPt-10130	3B	ns	0.048	3	ns	ns	-	0.05	ns	-
Xwmc777	3B	0.28	0.034	3	0.059	ns	-	ns	ns	-
wPt-5261	3B	ns	ns	-	0.0001	0.001	1,2	0.04	0.02	3,4
wPt-7301	3B	ns	ns	-	0.04	ns	2	ns	ns	-
wPt-11029	3B	ns	ns	-	0.0001	0.0001	1,2	0.06	0.02	4
wPt-0021	3B	ns	ns	-	0.0008	0.003	1,2	0.03	0.009	1,3,4
wPt-10537	3B	ns	ns	-	0.0001	0.0001	1,2	ns	ns	-
wPt-3342	3B	ns	ns	-	0.0001	0.0002	1,2	ns	ns	-
Xgwm247	3B	ns	ns	-	0.0001	0.0001	1,2	ns	ns	-
Xgwm181	3B	ns	ns	-	0.02	ns	-	ns	ns	-
Xgwm299	3B	ns	ns	-	0.01	ns	-	ns	ns	-
wPt-7158	3B	ns	ns	-	0.0001	0.0001	1,2	ns	0.0001	2,3
wPt-731500	3B	ns	ns	-	0.0001	0.0001	1,2	ns	0.0001	2,3
wPt-0668	3B	ns	ns	-	0.0001	0.0001	1,2	ns	ns	-
wPt-7614	3B	ns	ns	-	0.0001	0.0001	1,2	ns	0.0001	3
wPt-0324	3B	ns	ns	-	0.0001	0.0001	1,2	ns	ns	-
wPt-5562	3B	ns	ns	-	0.0001	0.0001	1,2	ns	ns	-
wPt-8959	3B	ns	ns	-	0.0001	0.0006	1,2	ns	ns	-
wPt-1046	4B	0.06	ns	-	ns	ns	-	0.0049	ns	-
wPt-732448	4B	ns	ns	-	ns	ns	-	0.00017	ns	-
wPt-2371	7A	0.0002	0.01	3	0.07	ns	-	0.06	ns	-
wPt-3702	7A	0.0008	0.02	2	ns	ns	-	ns	ns	-

[a] Individual markers were selected that were most closely associated with significant QTL across all three screening environments
[b] MSI = Marker x screen interaction term (significant at p<0.05)
[c] Screen = Screen number where the marker interaction was significant
[d] ns = not significant. P-value was not significant at p<0.05
