# Supplementary material for: Identification of quantitative trait loci (QTL) for resistance to Fusarium crown rot (Fusarium pseudograminearum) in multiple assay environments in the Pacific Northwestern US
Source: Theor Appl Genet. 2012 Feb 25;125(1):91–107. doi: 10.1007/s00122-012-1818-6 (PMC3351592; doi:10.1007/s00122-012-1818-6)
Supplement: Supplementary file 6 — Supplementary material 6 (PDF 106 kb) [file 122_2012_1818_MOESM6_ESM.pdf]

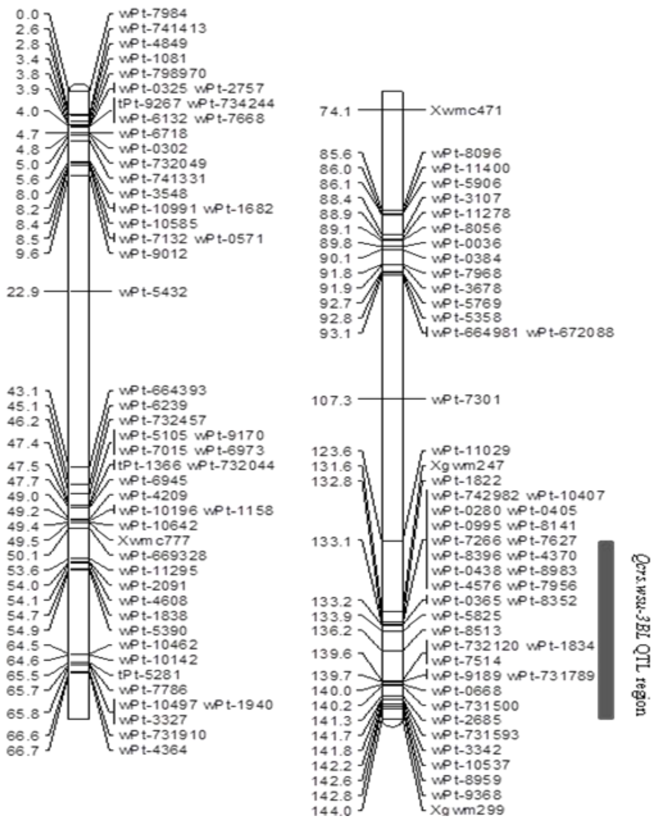

Online Resource 6. Consensus map of chromosome 3B constructed in JoinMap v4.0 by combining the Sunco/Macon and Sunco/Otis genetic linkage maps
