# Supplementary material for: Identification of quantitative trait loci (QTL) for resistance to Fusarium crown rot (Fusarium pseudograminearum) in multiple assay environments in the Pacific Northwestern US
Source: Theor Appl Genet. 2012 Feb 25;125(1):91–107. doi: 10.1007/s00122-012-1818-6 (PMC3351592; doi:10.1007/s00122-012-1818-6)
Supplement: Supplementary file 7 — Supplementary material 7 (PDF 10 kb) [file 122_2012_1818_MOESM7_ESM.pdf]

Online Resource 7. Results from the diversity panel analyzing the polymorphism of the markers *Xgwm247* and *Xgwm299* that flank the *Qcrs.wsu-3BL* QTL region

Genotype	Growth habit	Presence of FCR Resistance	*Xgwm247*	*Xgwm299*
Sunco	Spring	+	170	231
Macon	Spring	-	184	227
Otis	Spring	-	184	225
2-49	Spring	+	184	228
Alpowa	Spring	-	184	223
ARS970075-3	Winter	-	178	223
Bauermeister	Winter	-	184	227
Bitteroot	Winter	-	184	227
Bruehl	Winter	-	208	-
Brundage96	Winter	-	177	225
Chinese Spring	Spring	-	180	223
Chukar	Winter	-	130	225
CIMMYT-2	Facultative	-	184	227
Coda	Winter	-	182	225
Eddy	Winter	-	170	228
Eden	Spring	-	176	239
Eltan	Winter	-	184	227
Hartog	Spring	-	184	225
Finch	Winter	-	180	225
Finley	Winter	-	181	231
Gluyas early	Spring	+	184	217
Hank	Spring	-	184	227
Hollis	Spring	-	198	228
Kels	Spring	-	184	227
Lambert	Winter	-	184	225
Louise	Spring	-	176	139
Madsen	Winter	-	184	225
Masami	Spring	-	184	227
Opata	Spring	-	208	227
ORCF-101	Winter	-	192	225
ORCF-102	Winter	-	181	227
ORCF-103	Winter	-	184	227
Paladin	Winter	-	180	222
Pelsart	Winter	-	184	221
Penewawa	Spring	-	176	227
Puseas	Spring	-	184	227
Rod	Winter	-	178	222
Scarlet	Spring	-	178	227
Simon	Winter	-	180	225
JD	Spring	-	137	239
Stephens	Winter	-	184	225
VPM-1	Winter	-	192	225
Tara 2002	Spring	-	184	227
Tubbs 06	Winter	-	184	223
WB-528	Winter	-	184	227
WA8090	Spring	-	176	222
Xerpha	Winter	-	178	222
Zak	Spring	-	176	222
Gala	Spring	+	170	231
